# Frog α- and β-Ryanodine Receptors Provide Distinct Intracellular Ca^2+^ Signals in a Myogenic Cell Line

**DOI:** 10.1371/journal.pone.0011526

**Published:** 2010-07-12

**Authors:** Taku Kashiyama, Takashi Murayama, Erika Suzuki, Paul D. Allen, Yasuo Ogawa

**Affiliations:** 1 Department of Pharmacology, Juntendo University School of Medicine, Tokyo, Japan; 2 Department of Anesthesia, Perioperative and Pain Medicine, Brigham and Women's Hospital, Boston, Massachusetts, United States of America; University of Cincinnati, United States of America

## Abstract

**Background:**

In frog skeletal muscle, two ryanodine receptor (RyR) isoforms, α-RyR and β-RyR, are expressed in nearly equal amounts. However, the roles and significance of the two isoforms in excitation-contraction (E-C) coupling remains to be elucidated.

**Methodology/Principal Findings:**

In this study, we expressed either or both α-RyR and β-RyR in 1B5 RyR-deficient myotubes using the herpes simplex virus 1 helper-free amplicon system. Immunological characterizations revealed that α-RyR and β-RyR are appropriately expressed and targeted at the junctions in 1B5 myotubes. In Ca^2+^ imaging studies, each isoform exhibited caffeine-induced Ca^2+^ transients, an indicative of Ca^2+^-induced Ca^2+^ release (CICR). However, the fashion of Ca^2+^ release events was fundamentally different: α-RyR mediated graded and sustained Ca^2+^ release observed uniformly throughout the cytoplasm, whereas β-RyR supported all-or-none type regenerative Ca^2+^ oscillations and waves. α-RyR but not β-RyR exhibited Ca^2+^ transients triggered by membrane depolarization with high [K^+^]_o_ that were nifedipine-sensitive, indicating that only α-RyR mediates depolarization-induced Ca^2+^ release. Myotubes co-expressing α-RyR and β-RyR demonstrated high [K^+^]_o_-induced Ca^2+^ transients which were indistinguishable from those with myotubes expressing α-RyR alone. Furthermore, procaine did not affect the peak height of high [K^+^]_o_-induced Ca^2+^ transients, suggesting minor amplification of Ca^2+^ release by β-RyR via CICR in 1B5 myotubes.

**Conclusions/Significance:**

These findings suggest that α-RyR and β-RyR provide distinct intracellular Ca^2+^ signals in a myogenic cell line. These distinct properties may also occur in frog skeletal muscle and will be important for E-C coupling.

## Introduction

In vertebrate striated muscles, depolarization of transverse (T) tubule membranes triggers Ca^2+^ release from the sarcoplasmic reticulum (SR) in a process known as excitation-contraction (E-C) coupling. The Ca^2+^ release is mediated through the ryanodine receptor (RyR), a large homotetrameric channel complex (>2 MDa) in the SR membrane [1], [2]. In vertebrates there are three genetically distinct isoforms of RyR (RyR1–3). All the RyR channels exhibit Ca^2+^-induced Ca^2+^ release (CICR), in which Ca^2+^ itself activates the channel to release Ca^2+^ [3], [4]. In cardiac muscle, depolarization of the T-tubule activates the L-type Ca^2+^ channel (dihydropyridine receptor, DHPR) to enter extracellular Ca^2+^ into the cells. This entering Ca^2+^, in turn, triggers Ca^2+^ release from the RyR2 (predominant isoform in heart) via the CICR mechanism [5]. In skeletal muscle E-C coupling, in contrast, no extracellular Ca^2+^ entry is necessary. Ca^2+^ release from the RyR1 (predominant isoform in skeletal muscle) instead is triggered by conformational change of the voltage sensor in the DHPR upon depolarization of the T tubule (referred to as depolarization-induced Ca^2+^ release, DICR) [6], [7]. In DICR, some physical association between RyR and DHPR at the triad junction may be involved.

Adult mammalian skeletal muscles predominantly express RyR1. A small amount of RyR3 is also expressed in some adult muscles (diaphragm and soleus) and in most neonatal muscles [8], [9]. Functional studies with the RyR1-deficient ‘dyspedic’ mice revealed that RyR1 acts both as DICR and CICR channels, whereas RyR3 mediates CICR but not DICR [10], [11], [12]. Skeletal muscles of frog and many non-mammalian vertebrates have two isoforms of α-RyR and β-RyR, homologues of mammalian RyR1 and RyR3, respectively, in nearly equal amounts [13], [14]. β-RyR is proposed to be localized at the parajunctional position in the triad, which is adjacent to α-RyR at the junctional face [15]. In non-mammalian skeletal muscles, DICR is believed to be mediated by α-RyR because of its homology with mammalian RyR1 [16], [17], [18]. This is also suggested by the finding that some skeletal muscles of fish and chicken express α-RyR alone [19]. It has been proposed that the CICR activity of β-RyR may be >20-fold higher than that of α-RyR in frog skeletal muscle SR [20]. It was therefore hypothesized that the two isoforms may play distinct roles in Ca^2+^ release: DICR is mediated by α-RyR, whereas CICR is primarily supported by β-RyR [21]. However, there is no direct evidence for this hypothesis so far.

The RyR-deficient 1B5 myogenic cell line is a useful tool to investigate RyR isoforms [22]. They differentiate into multinucleated myotubes and express all of the normal key triadic proteins (e.g., DHPR complex, FKBP12, calsequestrin, junctin and triadin) but do not express any RyR isoform. Exogenous RyR can be targeted to the triad junction and restores E-C coupling in 1B5 myotubes [22], [23]. Thus, 1B5 cells provide a skeletal muscle context that is essential for proper function of RyRs. In this study, we expressed frog α-RyR and β-RyR in 1B5 myotubes to investigate physiological roles of the two RyR isoforms in muscle. We found that α-RyR but not β-RyR mediates DICR. In addition, the two isoforms demonstrated distinct Ca^2+^ release properties induced by caffeine. Our results suggest that the two RyR isoforms provide distinct intracellular Ca^2+^ signals in frog skeletal muscle.

## Methods

### Preparation of HSV-1 virus

cDNAs encoding the full-length bullfrog α-RyR and β-RyR (GenBank No. D21070 and D21071, respectively) were constructed from the partial cDNA clones [16] and then cloned into an HSV-1 amplicon vector pHSVprPUC [24]. The vector was co-transfected with DNA from the cosmid set into 2–2 cells to produce the HSV-1 virus using a helper virus-free packaging system [24]. Control virus lacking RyR gene was similarly produced with the amplicon vector without RyR cDNAs. Viral titers were determined immunohistochemically with BHK cells by counting the RyR-positive cells after infection of a fixed amount of the virus solution.

### Cell culture and viral infection

1B5 cells were cultured in growth medium (DMEM supplemented with 20% FCS, 100 µg/ml streptomycin sulphate, 100 units/ml penicillin-G) in 5% CO_2_ [22]. Cells were allowed to differentiate into myotubes by replacing the growth medium with differentiation medium (DMEM with 1% heat-inactivated horse serum, 100 µg/ml streptomycin sulphate, 100 units/ml penicillin-G) in 18% CO_2_ for 5 days. Myotubes were then infected with the HSV-1 virus for 2 h at 5×10^4^ virion particles per well and further cultured for 24 h. Mock infection was achieved using the control virus.

### Western blotting

1B5 myotubes cultured on 60 mm dishes were infected with the HSV-1 virus as described above. After 24 h infection myotubes were scraped from plates and centrifuged for 5 min at 1,000×g. The pellet was homogenized in phosphate buffered saline (PBS) containing 1% Triton X-100 and a cocktail of protease inhibitors, and the homogenate was centrifuged for 5 min at 10,000×g. The protein in the supernatant was processed by SDS-PAGE with a 2–12% gradient gel and transferred onto a PVDF membrane. RyRs were detected by polyclonal anti-RyR antibody which was raised against synthetic peptide corresponding to a sequence that was conserved among all known RyRs [25]. The positive bands were visualized using ECL Advance Western Blotting Detection Kit (GE Healthcare).

### Immunohistochemistry

1B5 cells cultured on a glass-bottom 35-mm plate were fixed in ice-cold methanol for 10 min at −20°C and then blocked for 1 hr in PBS supplemented with 1% BSA. Cells were incubated with primary antibodies overnight at 4°C. RyR was detected with monoclonal anti-RyR antibody 34C (Development Studies Hybridoma Bank, Iowa) [26], whereas DHPR was detected with polyclonal anti-DHPR α_1s_ subunit antibody that was raised against the synthetic peptide corresponding to 734–747 of bullfrog α_1s_ [27]. After washing three times for 10 min with PBS, cells were incubated for 1 h at room temperature with Alexa488-labeled goat anti-mouse IgG and Alexa594-labeled goat anti-rabbit IgG (Molecular Probes). After successive washing with PBS, the fluorescence signal was viewed on a laser scanning confocal microscope (Oz system; Noran Instruments) equipped with an Argon Krypton Ion Laser System (488 and 568 nm excitation).

### Ca^2+^ imaging

For Ca^2+^ imaging, 1B5 myotubes were cultured as described above on a 96-well clear bottom plate (Corning Costar) that had been treated with Matrigel (BD Bioscience). After 24 h infection the myotubes were loaded with 5 µM fluo-4 AM (Molecular Probes) for 30 min at 37°C in a bath solution (130 mM NaCl, 4.7 mM KCl, 1.2 mM MgSO_4_, 1.9 mM CaCl_2_, 1.2 mM KH_2_PO_4_, 11 mM glucose, and 20 mM HEPES, pH 7.2 adjusted with NaOH). The cells were set on an inverted microscope equipped with a fluorescence imaging system (AquaCosmos, Hamamatsu, Japan). Fluo-4 was excited at 488 nm using a xenon arc lamp with a monochrometer and fluorescence emission through a band-pass filter (535/45) was recorded with a CCD camera using a 10× objective lens (AquaCosmos, Hamamatsu). All measurements were carried out at room temperature (22–25°C). The ratio signals (F/F_0_) of individual myotubes were determined. To stimulate the myotubes, solutions (1 ml) were perfused into the wells by suction. Solutions with different concentrations of K^+^ (high [K^+^]_o_, 20–130 mM) were made in order to keep [K^+^]× [Cl^−^] constant by replacing chloride with methanesulfonate. Caffeine (0.2–20 mM) was dissolved in the bath solution. CaCl_2_ was omitted from the bath solution prior to and during stimuli to prevent Ca^2+^ influx via the sarcolemma.

## Results

### Expression of frog RyR isoforms in differentiated 1B5 myotubes

**Figure 1:**
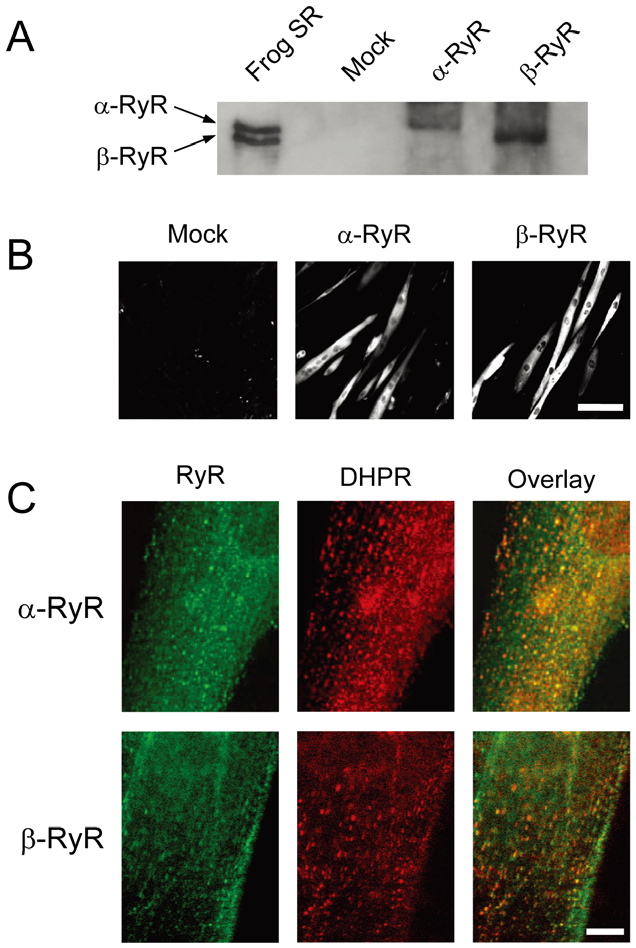
Expression of α-RyR and β-RyR in differentiated 1B5 myotubes. 1B5 cells were differentiated into myotubes for five days, infected with HSV virions containing either frog α-RyR or β-RyR cDNA, and assayed 24 h after infection. **A**. Expression of RyRs was detected using polyclonal pan-specific anti-RyR antibody in total lysate from myotubes transduced with either mock, α-RyR or β-RyR virions. A single band corresponding to α-RyR and β-RyR in the frog SR vesicles was detected in the infected myotubes. No bands were detected in mock-infected myotubes. **B**. Myotubes were labeled with monoclonal anti-RyR antibody (34C). RyR immunoreactivity was detected in the cytoplasm of myotubes transduced with α-RyR and β-RyR, but not in the mock-infected myotubes. Scale bar, 100 µm. **C**. Myotubes were labeled with 34C (green) and polyclonal anti-DHPR α_1S_ subunit antibody (red). RyRs were localized as discrete foci near the cell surface, which were co-localized with the DHPR in both α-RyR and β-RyR transduced cultures. Scale bar, 10 µm.

Frog RyR isoforms were expressed in 1B5 myotubes by transducing them with helper free HSV-1 amplicon virions (5×10^5^ infectious units/ml) [24] containing either bullfrog α-RyR or β-RyR cDNA. A single band with high molecular mass was detected by anti-RyR antibody in total lysate from myotubes transduced with α-RyR or β-RyR virions, whereas no band was seen in the mock-infected myotubes (**Fig. 1A**). The mobility of each band corresponds to that of the native proteins from frog skeletal muscle SR, suggesting expression of full-length proteins. Immunohistochemistry with RyR specific antibody (34C) showed that myotubes were labeled by the antibody throughout the cytoplasm except for the nuclei (**Fig. 1B**). Closer examinations revealed that α-RyR and β-RyR exist as discrete foci that were located close to the cell surface (**Fig. 1C**). These foci appear to be co-localized with the DHPR (**Fig. 1C**). Taken together, these findings indicate that frog α-RyR and β-RyR are properly expressed and targeted at the triad junctions in 1B5 myotubes.

### Depolarization-induced and caffeine-induced Ca^2+^ transients of 1B5 myotubes expressing frog RyR isoforms

DICR and CICR activity of frog RyR isoforms were investigated by intracellular Ca^2+^ ([Ca^2+^]_i_) measurements of individual cells using fluo-4 (see Materials and Methods). DICR activity was evaluated from Ca^2+^ release induced by high potassium in the bath solution (high [K^+^]_o_), which triggers membrane depolarization. CICR activity was assessed by caffeine-induced Ca^2+^ release. To exclude the possibility of Ca^2+^ influx via the sarcolemma, Ca^2+^ was omitted from the bath solution prior to and during stimuli.

**Figure 2:**
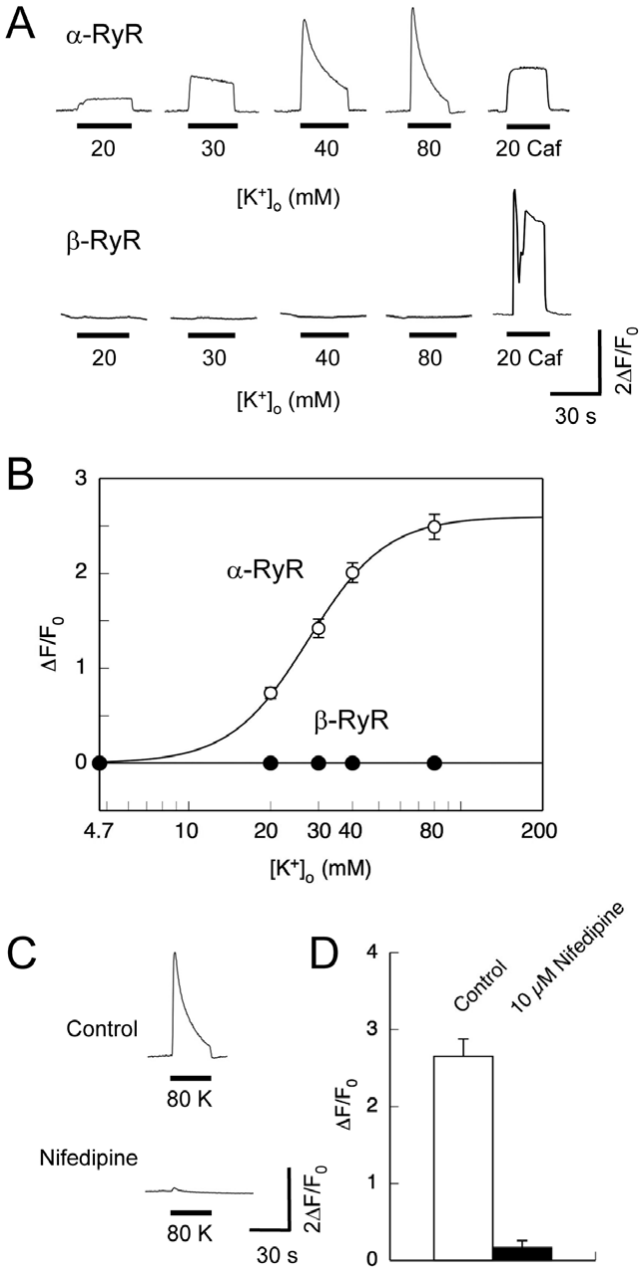
Depolarization-induced Ca^2+^ release. Intracellular Ca^2+^ ([Ca^2+^]_i_) of 1B5 myotubes transduced with either α-RyR or β-RyR virions was imaged using fluo-4 as described in Materials and Methods. Cells were exposed for 30 sec to varied high [K^+^]_o_ solutions (with the constant [K^+^]×[Cl^−^] product) to trigger DICR, and finally to 20 mM caffeine to confirm the functional expression of RyR. Ca^2+^ in the bath solution was omitted prior to and during stimuli to prevent Ca^2+^ influx. **A**. Representative traces of Ca^2+^ transients of individual cells by [K^+^]_o_ and caffeine. **B**. Averaged maximal change in fluo-4 fluorescence (ΔF/F_0_) was plotted against [K^+^]_o_ concentration. Values are expressed as mean ± SE (n = 112 for α-RyR and 18 for β-RyR). α-RyR but not β-RyR exhibited Ca^2+^ transients induced by high [K^+^]_o_. **C**. A representative trace of Ca^2+^ transients of myotubes expressing α-RyR stimulated with 80 mM [K^+^]_o_ before and after treatment with 10 µM nifedipine. **D**. Averaged maximal change in fluo-4 fluorescence (ΔF/F_0_) was plotted with or without 10 µM nifedipine. Values are expressed as mean ± SE (n = 15). Nifedipine inhibited high [K^+^]_o_-induced Ca^2+^ transients.

In mock-infected myotubes, neither high [K^+^]_o_ (up to 80 mM) nor caffeine (up to 20 mM) triggered Ca^2+^ transients, corresponding to a lack of detected RyRs (data not shown). The efficiency of HSV virion transduction was ∼80%. All myotubes successfully transduced with α-RyR virions exhibited Ca^2+^ transients induced by increasing [K^+^]_o_ in a dose-dependent manner and seemed to reach a plateau at 80 mM [K^+^]_o_ (**Fig. 2A, 2B**). They also had Ca^2+^ transients after perfusion with 20 mM caffeine. By contrast, the myotubes transduced with β-RyR responded to caffeine, but no myotubes were responsive to high [K^+^]_o_ up to 80 mM. Lack of high [K^+^]_o_ response in β-RyR does not stem from improper expression or targeting of the isoform, because it was expressed as a full-length protein and co-localized with the DHPR (see **Fig. 1**). The [K^+^]_o_ dependence of Ca^2+^ transients by α-RyR demonstrated that the EC_50_ value of [K^+^]_o_ was about 28 mM (**Fig. 2B**), which is in good agreement with the values determined by high [K^+^]_o_-induced tension development in intact skeletal muscle fibers [28], [29]. The Increasing [K^+^]_o_ also accelerated the time-dependent decline in [Ca^2+^]_i_ which can be explained by inactivation of the voltage sensor (**Fig. 2A**). The high [K^+^]_o_-induced Ca^2+^ transients were strongly inhibited by 10 µM nifedipine, which selectively blocks the DHPR voltage sensor (**Fig. 2C, 2D**). This is consistent with the properties of DICR in frog and mammalian skeletal muscles [6], [7] and indicates that frog α-RyR but not β-RyR mediates DICR that is coupled to and controlled by endogenous DHPRs in 1B5 myotubes.

**Figure 3:**
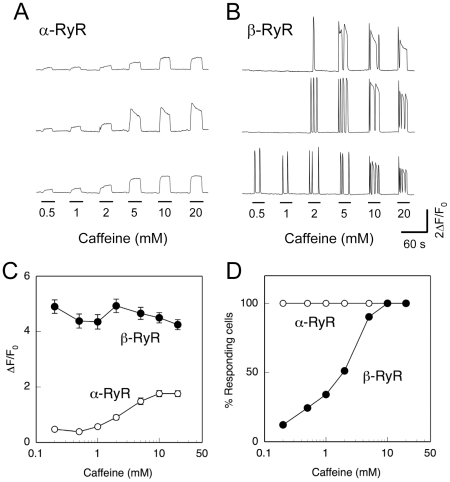
Caffeine-induced Ca^2+^ release. **A, B**. Myotubes expressing α-RyR (**A**) or β-RyR (**B**) were stimulated by varied concentrations (0.2–20 mM) of caffeine. Traces of three representative cells are shown. Sustained Ca^2+^ transients with no or only slight decline were observed with myotubes expressing α-RyR, whereas Ca^2+^ oscillations with rapid rise and fall phases were seen with myotubes expressing β-RyR. **C**. Average maximal changes in fluo-4 fluorescence (ΔF/F0) of responding cells plotted against caffeine concentration (mean ± SE, n = 34 and 41 for α-RyR and β-RyR, respectively). Open circles, α-RyR; filled circles, β-RyR. In cells expressing α-RyR, Ca^2+^ transient amplitudes increased with increasing caffeine concentrations, whereas the responses in cells expressing β-RyR after reaching threshold were independent of caffeine concentration. **D**. % myotubes responding to each caffeine concentration. Open circles, α-RyR (n = 34); filled circles, β-RyR (n = 41). All the myotubes expressing α-RyR responded to caffeine with graded magnitude, whereas β-RyR myotubes showed all-or-none responses with increasing fractions of responding cells as the caffeine concentration increased.

Fig. 3 demonstrates caffeine-induced Ca^2+^ transients of myotubes expressing α-RyR or β-RyR. Myotubes expressing α-RyR exhibited dose-dependent Ca^2+^ transients in response to caffeine with only a slight decline in signal during a 30 sec exposure (**Fig. 3A**). The peak amplitude of the caffeine-induced Ca^2+^ release increased with caffeine dose and saturated around 10 mM (**Fig. 3A, 3C**). Myotubes expressing β-RyR exhibited Ca^2+^ oscillations with rapid rise and fall phases (like a burst of spikes) during exposure to caffeine (0.2–20 mM) (**Fig. 3B**). Notably, there are large cell-to-cell variations in threshold concentrations for caffeine. The individual Ca^2+^ transients tended to be longer in duration at a higher caffeine dose, and some cells exhibited long-lasting (>10 sec) Ca^2+^ transients when the caffeine concentration was 5 mM or higher. The peak amplitude of Ca^2+^ oscillations was constantly high in an all-or-none fashion, irrespective of caffeine dose (**Fig. 3C**). The peak amplitude of β-RyR is greater than the maximum response of α-RyR, which shows a sustained Ca^2+^ release (**Fig. 3B, 3C**). The fraction of responding cells increased with caffeine dose (**Fig. 3D**). This is in marked contrast to myotubes expressing α-RyR, where all expressing myotubes responded to the caffeine doses examined in a graded manner (**Fig. 3A, 3D**).

### Spatiotemporal properties of Ca^2+^ transients of 1B5 myotubes expressing frog RyR isoforms

**Figure 4:**
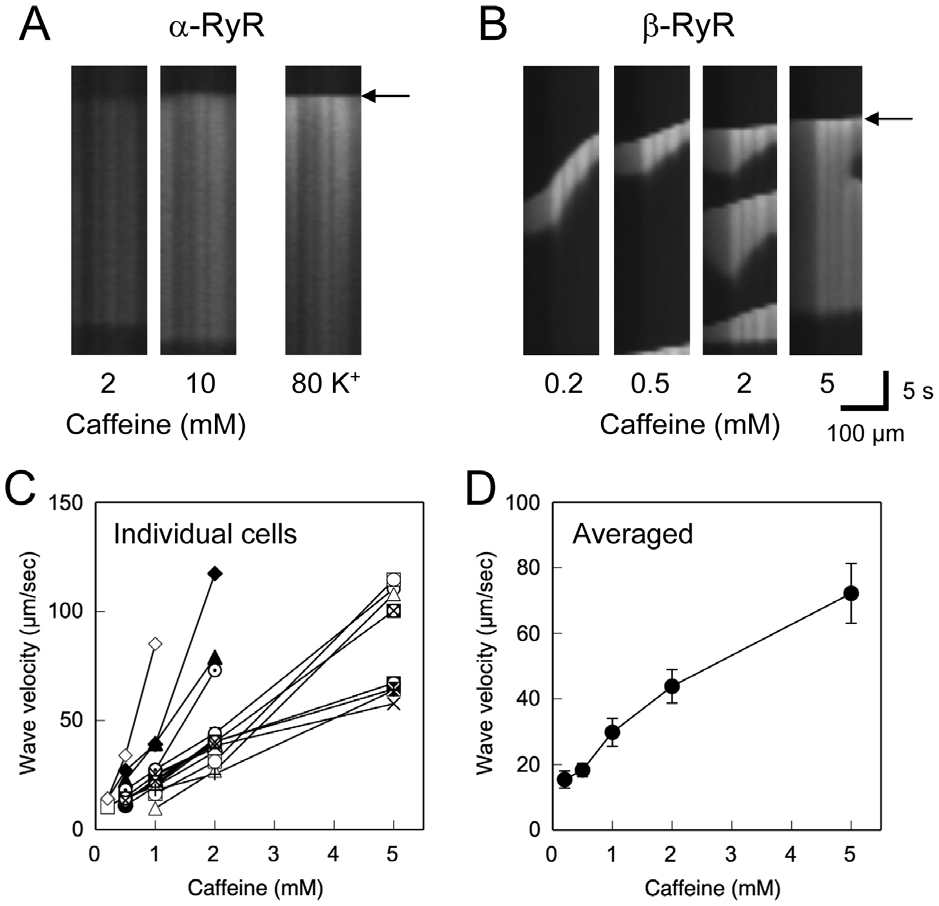
Spatiotemporal properties of Ca^2+^ release. **A**, **B**. Line scan analysis of Ca^2+^ transients of 1B5 myotubes expressing α-RyR (**A**) and β-RyR (**B**) induced by caffeine or high [K^+^]_o_. Scan lines were set on along their longitudinal axes of myotubes. Ca^2+^ transients within myotubes expressing α-RyR uniformly occurred instantaneously throughout the cells, whereas those of myotubes expressing β-RyR occurred at specific sites and propagated in waves within the cell. **C**, **D**. Wave velocity of individual cells expressing β-RyR (**C**) and its average (**D**) was plotted against caffeine dose. Wave velocity increased with caffeine dose (mean±SE, n = 15).

The above results suggest a substantial difference in Ca^2+^ release properties of α-RyR and β-RyR. To deepen understanding of the Ca^2+^ release mechanism, we investigated spatiotemporal properties of Ca^2+^ transients of the two RyR isoforms. Caffeine-induced Ca^2+^ transients in myotubes expressing α-RyR occurred uniformly within the cell (**Movie S1**). Line scan analysis along their longitudinal axes of the myotubes clearly demonstrates a uniform increase in Ca^2+^ throughout the cell (**Fig. 4A**). Similar responses were also observed when myotubes were stimulated with high [K^+^]_o_ (**Fig. 4A** **, Movie S2**). In contrast, myotubes expressing β-RyR exhibited Ca^2+^ waves, which occurred at specific sites and propagated within cells (**Fig. 4B** **, Movie S3**). At a higher caffeine doses in cells expressing β-RyR (≥5 mM), the initial Ca^2+^ transients occurred almost instantaneously throughout the myotubes, followed by the repetitive Ca^2+^ waves. Notably, velocity of the Ca^2+^ waves increased with caffeine dose, although substantial cell-to-cell variations were apparent (**Figs. 4C**). Averaged velocities of Ca^2+^ waves in myotubes expressing β-RyR were 15±3 µm/sec at 0.2 mM caffeine, 18±2 µm/sec at 0.5 mM caffeine, 30±4 µm/sec at 1 mM caffeine, 44±5 µm/sec at 2 mM caffeine, and 72±9 µm/sec at 5 mM caffeine (**Fig. 4D**). The velocities are similar with those of the caffeine-induced Ca^2+^ waves of frog skeletal muscle [30] as well as the other cell types [31], [32]. These findings suggest that α-RyR and β-RyR have distinct spatiotemporal properties of Ca^2+^ release in 1B5 myotubes.

### Depolarization-induced and caffeine-induced Ca^2+^ transients of 1B5 myotubes expressing both RyR isoforms

**Figure 5:**
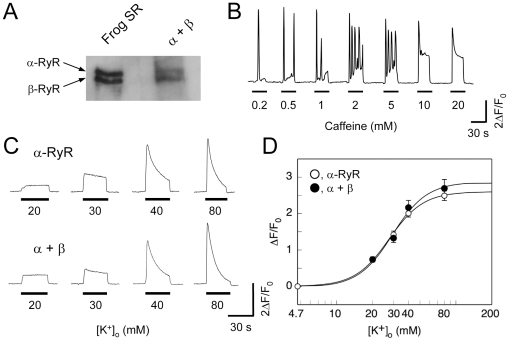
Ca^2+^ release properties of 1B5 myotubes co-expressing α-RyR and β-RyR. **A**. Immunoblot of total lysate from 1B5 myotubes co-expressing α-RyR and β-RyR showing two bands corresponding in size to those observed in preparations of frog SR. **B**. A representative trace of Ca^2+^ transients induced by varied concentrations of caffeine. The responses of cells co-expressing both isoforms appeared to be additive and independent of each other: uniform and sustained Ca^2+^ release characteristic of α-RyR and Ca^2+^ oscillations characteristic β-RyR were both observed in these cells. **C**. Representative Ca^2+^ transients of myotubes expressing either α-RyR alone (upper traces) or α-RyR and β-RyR expressed together (lower traces) induced by increasing concentrations of [K^+^]_o_. **D**. [K^+^]_o_ dependences of the Ca^2+^ transients of myotubes expressing α-RyR (open circles) and co-expressing α-RyR and β-RyR (closed circles). (mean ± SE, n = 112 for α-RyR and 121 for α-RyR and β-RyR). There was no statistical difference between the two groups at any [K^+^]_o_ concentration examined.

It has been proposed that CICR of β-RyR may serve to amplify the Ca^2+^ signals of DICR by α-RyR [33]. To assess this possibility, we expressed both isoforms in 1B5 myotubes and examined their Ca^2+^ release properties. 1B5 myotubes were transduced with a mixture of α-RyR and β-RyR virions. Immunoblotting of total lysate of the infected myotubes revealed that α-RyR and β-RyR were expressed in nearly equal amounts (**Fig. 5A**). These myotubes exhibited caffeine-induced Ca^2+^ transients that consisted of Ca^2+^ oscillations and the sustained Ca^2+^ rise, which correspond to the combined phenotype of cells expressing the individual isoforms (**Fig. 5B**). This confirmed functional expression of both α-RyR and β-RyR in the myotubes. The two isoforms were independent of each other in their Ca^2+^ release fashion. The overall features of high [K^+^]_o_-induced Ca^2+^ transients in myotubes expressing the two isoforms, however, were similar to those with myotubes expressing α-RyR alone (**Fig. 5C**). Neither Ca^2+^ oscillations nor waves observed during the stimuli. The averaged amplitude and [K^+^]_o_ dependence of the Ca^2+^ transients were not significantly different from those of myotubes expressing α-RyR alone (**Fig. 5D**). Thus, co-expression of β-RyR did not affect DICR of α-RyR.

**Figure 6:**
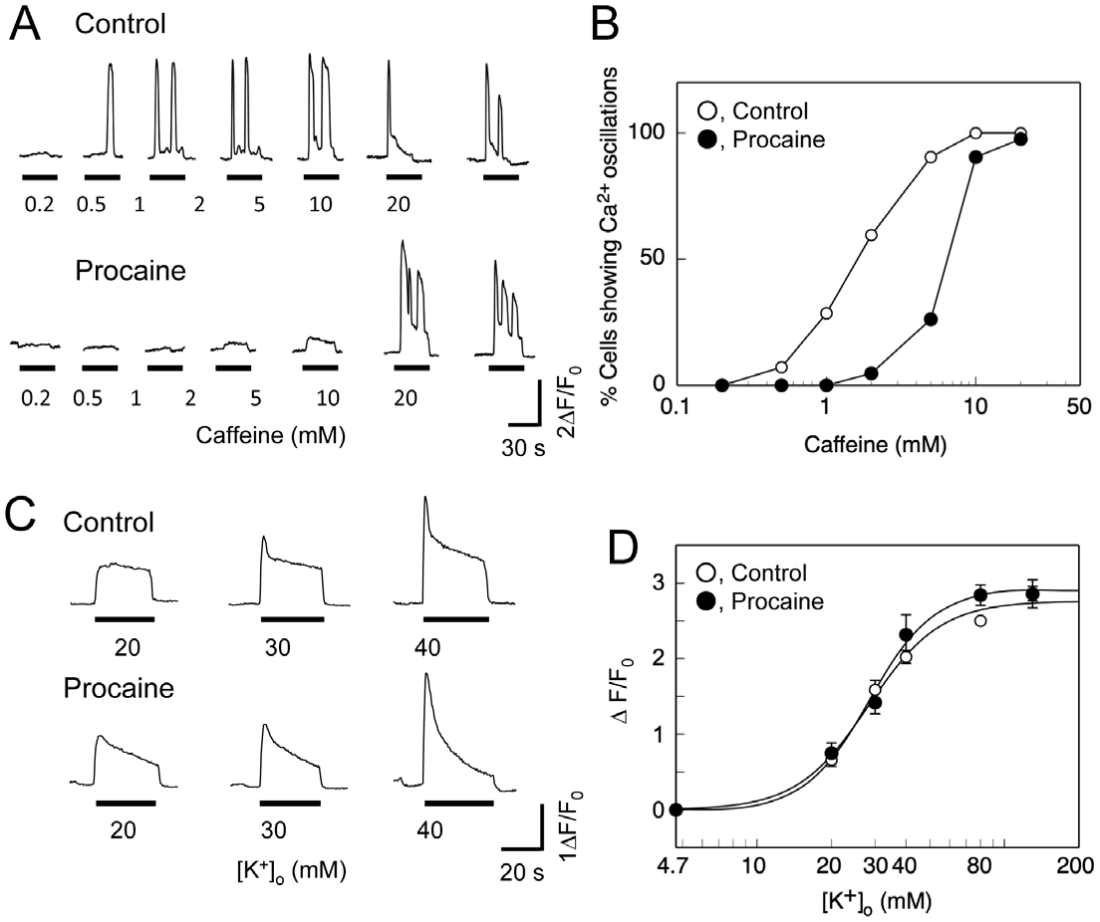
Effect of procaine on Ca^2+^ release properties of 1B5 myotubes co-expressing α-RyR and β-RyR. **A**. Representative Ca^2+^ transients induced by increasing concentrations of caffeine in the absence (Control) and presence (Procaine) of 10 mM procaine. Procaine abolished Ca^2+^ oscillations caused by exposure to lower concentrations (<5 mM) of caffeine. **B**. The percentage of myotubes showing Ca^2+^ oscillations at each caffeine concentration tested was plotted in reference to the number of cells responding in the presence of 20 mM caffeine. Open circles, control; filled circles, 10 mM procaine (n = 42). Procaine reduced the caffeine sensitivity. **C**. Representative traces of Ca^2+^ transients of myotubes induced by increasing [K^+^]_o_ in the absence (Control) and presence (Procaine) of 10 mM Procaine. **D**. Dependence of the amplitude of the Ca^2+^ transient in response to in exposure to increasing [K^+^]_o_ in the absence (open circles) and presence (filled circles) of 10 mM Procaine. Values represent the mean ± SE (n = 42). No statistical difference was found between the two groups at all the [K^+^]_o_ concentrations examined.

To further evaluate the role of β-RyR on DICR, we examined the effect of procaine, an inhibitor of CICR, on the Ca^2+^ transients of myotubes co-expressing α-RyR and β-RyR. Procaine (10 mM) abolished the Ca^2+^ oscillations induced by lower concentrations (≦5 mM) of caffeine (**Fig. 6A**) and reduced the caffeine sensitivity by nearly fivefold (**Fig. 6B**), suggesting that CICR via β-RyR is strongly inhibited by procaine. The sustained Ca^2+^ rises probably caused by α-RyR were also reduced by procaine (**Fig. 6A**). Similar inhibition of caffeine-induced Ca^2+^ release by procaine was observed with myotubes expressing α-RyR or β-RyR alone (data not shown). These findings suggest that procaine effectively inhibited CICR of α-RyR and β-RyR. In contrast, procaine did not affect the peak value of high [K^+^]_o_-induced Ca^2+^ transients (**Fig. 6C, 6D**) but it did accelerate the subsequent decline of the Ca^2+^ transients (**Fig. 6C**). This is consistent with the reports that procaine did not inhibit activation of the DHPR voltage sensor and consequent Ca^2+^ release, but it accelerated the inactivation process [34], [35]. Taken together, these findings suggest that CICR by β-RyR may make only a minor contribution to Ca^2+^ signals of DICR on membrane depolarization in 1B5 myotubes.

## Discussion

In this study, we investigated the functions and roles of two RyR isoforms (α-RyR and β-RyR) in frog skeletal muscle by expressing them in 1B5 myotubes. 1B5 myotubes have been derived from mouse and successfully used for functional reconstitution of mammalian RyR isoforms [12], [22], [23]. We here demonstrated that both α-RyR and β-RyR were expressed as full-length proteins and detected as discrete foci that were co-localized with the DHPR in the myotubes (**Fig. 1**). Both constructs formed functional Ca^2+^ release channels that were activated by caffeine (**Fig. 3**). Furthermore, α-RyR exhibited high [K^+^]_o_-induced Ca^2+^ release which is controlled by the endogenous DHPR (**Fig. 2**). This is not surprising, because the amino acid sequences of the channel forming α_1S_ [36] and other auxiliary subunits (β_1_, α_2_/δ, andγ) (E. Suzuki and Y. Ogawa, GenBank accession No. AB043621–7) of bullfrog skeletal muscle DHPR are sufficiently homologous to those of the mammalian skeletal muscle counterparts that species differences should not have posed a problem. Thus, 1B5 myotubes provide a skeletal muscle context that is sufficient for proper function of frog α-RyR and β-RyR.

### Differential Ca^2+^ release properties of two RyR isoforms

Our results indicate several functional differences in Ca^2+^ release properties between frog α-RyR and β-RyR. First, α-RyR but not β-RyR mediates high [K^+^]_o_-induced Ca^2+^ release, i.e., DICR (**Fig. 2**). This view is common to RyR homologues of other vertebrate skeletal muscles [12], [37], [38]. Second, the fashion of caffeine-induced Ca^2+^ release events is fundamentally different between the two isoforms: cells expressing α-RyR have uniform and sustained Ca^2+^ release in a graded manner, whereas cells expressing β-RyR have regenerative Ca^2+^ release events (Ca^2+^ oscillations and waves) in an all-or-none fashion (**Fig. 3** **,** **4**). These differences may be due to intrinsic Ca^2+^ release properties of the two RyR isoforms, but is not to secondary differences (e.g., [Ca^2+^]_i_ or [Ca^2+^]_SR_) induced by expression of each isoform, because a combined phenotype of caffeine-induced Ca^2+^ release was observed when the two isoforms were co-expressed (**Fig. 5**). We have demonstrated previously that [^3^H]ryanodine binding of β-RyR is more than 20-fold higher than that of α-RyR in frog skeletal muscle SR, indicating greater CICR activity of β-RyR [20]. This might be partly responsible for occurrence of regenerative Ca^2+^ release events by β-RyR. Further studies would clarify the underlying mechanisms of these differences.

Ca^2+^ sparks are discrete and localized elevation of Ca^2+^ via the RyR channels [39], [40]. Based on the facts that Ca^2+^ sparks are readily detectable in frog muscle, but hardly detected in mammalian muscle [41], [42], it is hypothesized that β-RyR or RyR3 may produce the Ca^2+^ sparks. Instead, “ridge” and “ember”, a long-lasting events of steady amplitude, have also been detected in frog [43] and mammalian [44] skeletal muscles, which are believed to represent the Ca^2+^ release operated directly by voltage sensors. The regenerative and self-terminating fashion of Ca^2+^ release events by β-RyR is consistent with the properties of Ca^2+^ sparks, whereas the ember-type Ca^2+^ release could be explained by graded and sustained Ca^2+^ release by α-RyR.

### Roles of two RyR isoforms in frog skeletal muscle

We demonstrated here that DICR is exclusively mediated by α-RyR. This clearly indicates that α-RyR is critical for E-C coupling in frog skeletal muscle. What is a role of β-RyR? It has been proposed that β-RyR might function as an amplifier of the Ca^2+^ signals by the CICR mechanism [33]. Contribution of CICR to physiological Ca^2+^ release in frog skeletal muscle, however, has been challenged by Endo [3], [45] and us [21], [46]. We here demonstrated that global Ca^2+^ transients induced by high [K^+^]_o_ were not affected by co-expression of β-RyR (**Fig. 5**). In addition, procaine had no effect on high [K^+^]_o_-induced Ca^2+^ transients of myotubes co-expressing α-RyR and β-RyR (**Fig. 6**). Taken together, these findings suggest that CICR by β-RyR may make only a minor contribution to Ca^2+^ signals of DICR on membrane depolarization in 1B5 myotubes.

Recently, Pouvreau et al. [47] have expressed exogenous mammalian RyR3 in adult mouse skeletal muscle fibers by electroporation of its cDNA. They found that RyR3-transfected fibers exhibited abundant voltage-activated Ca^2+^ sparks that were not observed with non-transfected or RyR1-transfected fibers, suggesting the amplification of Ca^2+^ release by RyR3. Legrand et al. [48] conducted similar experiments where exogenous mammalian RyR3 was expressed, but the results were totally opposite of those found by Pouvreau: the RyR3-transfected fibers exhibited no voltage-activated Ca^2+^ sparks, although spontaneous Ca^2+^ release events were frequently observed. This is consistent with our conclusion that β-RyR makes only a minor contribution to DICR. The reason for the differences between the two studies remains unclear.

Luttgau and Oetliker [49] reported that the threshold concentration of caffeine contracture was 1–2 mM with adult frog skeletal muscle and that caffeine at 2–4 mM caused occasional oscillations of tension, although it gave rise to sustained contracture at 5 mM or higher. On the basis of our findings presented here (**Fig. 3** **,** **5**), greater contribution of Ca^2+^ release through β-RyR than that through α-RyR would be a plausible explanation for tension oscillation in the presence of 2–4 mM caffeine. On the other hand, the characteristic disposition of α-RyR and β-RyR on the junctional face of the SR [15] might have to be kept in mind for interpretation of results with adult frog skeletal muscle. This arrangement may be critically important for effective activation of CICR of β-RyR [33]. In the present study we did not perform ultrastructural analysis of the arrangement of the two RyR isoforms in 1B5 myotubes. However, we demonstrated that each isoform was properly targeted to the junctions as clearly as the two studies of skeletal muscles expressing exogenous RyR3 showed [47], [48]. Further studies will be necessary to conclude the function and contribution of β-RyR to E-C coupling in frog skeletal muscle.

### Differential effects of procaine on CICR and DICR

Procaine is a well-known inhibitor of CICR. Its effect on DICR, however, is controversial [34], [35], [50], [51]. The results presented here indicate that procaine has no or only weak effect on DICR under conditions free from inactivation of E-C coupling, but it inhibits DICR during the inactivated state. Thus results using procaine would depend on experimental conditions: method of stimulation or activation, the size or mass of biological specimen, the type of preparation, and experimental history.

### Conclusion

In conclusion, our results suggest that the two frog RyR isoforms provide distinct intracellular Ca^2+^ signals in 1B5 myotubes. These distinct properties may also occur in frog skeletal muscle and will be important for E-C coupling.

## Supporting Information

Movie S1 Caffeine-induced Ca^2+^ transients of myotubes expressing α-RyR. Myotubes were stimulated by 10 mM caffeine. Images were captured at every 900 ms. The movie is 5 times faster in speed than real time.
(0.02 MB MOV)

Movie S2 [K^+^]_o_-induced Ca^2+^ transients of myotubes expressing α-RyR. Myotubes were stimulated by 80 mM [K^+^]_o_. Images were captured at every 300 ms. The movie is 5 times faster in speed than real time.
(0.05 MB MOV)

Movie S3 Caffeine-induced Ca^2+^ transients of myotubes expressing β-RyR. Myotubes were stimulated by 10 mM caffeine. Images were captured at every 900 ms. The movie is 5 times faster in speed than real time.
(0.04 MB MOV)
